# Virologic characteristics of SARS-CoV-2 infection across evolving Omicron subvariants

**DOI:** 10.1172/jci.insight.192228

**Published:** 2025-09-09

**Authors:** Julie Boucau, Owen T. Glover, Caitlin Marino, Gregory E. Edelstein, Manish C. Choudhary, Yijia Li, Brooke M. Leeman, Zahra Reynolds, Karry Su, Dessie Tien, Chase B. Mandell, Eliza Passell, Andrew Alexandrescu, Emory Abar, Mamadou Barry, Dibya Ghimire, Tammy D. Vyas, Jatin M. Vyas, Jacob E. Lemieux, Jonathan Z. Li, Mark J. Siedner, Amy K. Barczak

**Affiliations:** 1 Ragon Institute of Mass General Brigham, Cambridge, Massachusetts, USA.; 2 Brigham and Women’s Hospital, Boston, Massachusetts, USA.; 3 University of Pittsburgh Medical Center, Pittsburgh, Pennsylvania, USA.; 4 Massachusetts General Hospital, Boston, Massachusetts, USA.; 5 Harvard Medical School, Boston, Massachusetts, USA.; 6 Broad Institute, Cambridge, Massachusetts, USA.; 7 Africa Health Research Institute, KwaZulu-Natal, South Africa.

**Keywords:** Clinical Research, Infectious disease, COVID-19

## Abstract

**BACKGROUND:**

SARS-CoV-2 has evolved subvariants since the emergence of the Omicron variant in 2021. Whether these changes impact viral shedding and transmissibility is not known.

**METHODS:**

POSITIVES is a prospective longitudinal cohort of individuals with mild SARS-CoV-2 infection. Ambulatory, immunocompetent participants who did not receive antivirals self-administered 6 anterior nasal swabs over 15 days. Samples were analyzed by qPCR to quantify viral RNA, semiquantitative viral culture to detect shedding of replication-competent virus, and whole-genome sequencing to classify subvariants. Our predictor of interest was Omicron subvariants: BA.1x, BA.2x, BA.4/5x, XBB.x, and JN.x. Outcomes included RNA levels and duration of shedding replication-competent virus. We additionally explored whether symptoms are a valid marker for ending isolation.

**RESULTS:**

The median peak nasal SARS-CoV-2 RNA (6.0–6.3 log_10_ RNA copies/mL), median days to peak RNA (4–5 days), median days to undetectable viral RNA (12–14 days), and median days to negative viral culture (4–8 days) were similar across Omicron subvariants. Number and duration of symptoms were also similar. For all subvariants, a sizeable percentage (range 27.5%–56.0%) shed replication-competent virus after fever resolution and improvement of symptoms.

**CONCLUSION:**

Despite ongoing viral evolution, key aspects of viral dynamics of SARS-CoV-2 infection, including the duration of shedding replication-competent virus, have not substantially changed across Omicron subvariants. Replication-competent shedding of these subvariants is detected for a large proportion of people who meet criteria for ending isolation.

**FUNDING:**

NIH (U19 AI110818, R01 AI176287, K24 HL166024), the Massachusetts Consortium on Pathogen Readiness, and the Massachusetts General Hospital Department of Medicine.

## Abstract 2

The course of COVID-19 infection, including duration of likely infectiousness and symptoms, has not changed across Omicron variants. Symptoms, which form the basis for current public health guidance, are not a reliable reflection of likely infectiousness.

## Introduction

Although the morbidity and mortality associated with SARS-CoV-2 infection has decreased substantially since the earliest days of the pandemic, SARS-CoV-2 remains a leading cause of upper respiratory infection and a cause of substantial global mortality (1). Over the last 5 years, SARS-CoV-2, the virus that causes COVID-19, has repeatedly evolved, with the emergence of new variants that are phenotypically distinct from previous variants (2–4). At the same time, vaccination and native infection have increased population immunity to SARS-CoV-2. Yet, there are limited data about whether and how SARS-CoV-2 virology has changed during this time. Furthermore, while isolation guidelines have shifted to become symptom-focused with reduced periods of isolation duration (5), whether symptoms accurately reflect virologic features of infection or active viral shedding is not well reported.

## Results

### Cohort characteristics.

We enrolled participants in a prospective longitudinal cohort of ambulatory, immunocompetent individuals with SARS-CoV-2 infection (Post-Vaccination Viral Characteristics Study, POSITIVES) (6–14) to compare virologic characteristics across Omicron subvariants. Inclusion criteria for this study included outpatient status, no immunosuppression, no antiviral treatment, at least 4 samples collected, and sequencing confirmation of the infecting strain as Omicron (Supplemental Figure 1; supplemental material available online with this article; https://doi.org/10.1172/jci.insight.192228DS1). Out of 300 enrollees in POSITIVES from December 2021 to September 2024 eligible for inclusion, 160 were excluded for collecting less than 4 samples (*n* = 11), receipt of antiviral treatment (*n* = 122), or an infecting variant other than Omicron (*n* = 27), resulting in an analytic cohort of 140 participants (Supplemental Figure 1). Participants self-collected an average of 6 nasal swabs over 15 days. Samples were analyzed by qPCR to quantify viral RNA, semiquantitative viral culture to assess the presence of replication-competent virus, and whole-genome sequencing to identify the infecting SARS-CoV-2 subvariant.

### Subvariant groups.

**Figure 1:**
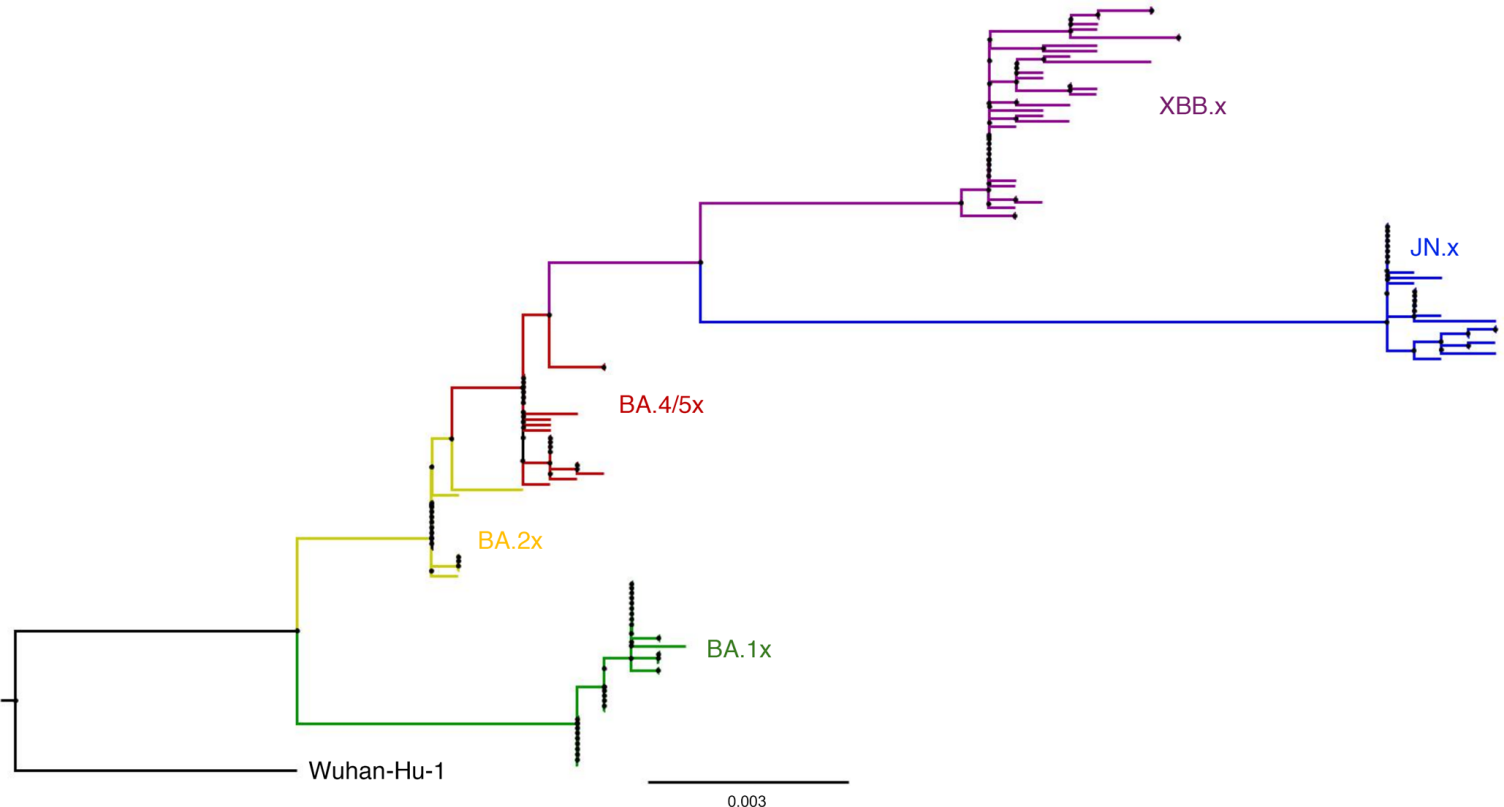
Phylogenetic tree representing the infecting Omicron subvariants in this study based on spike gene sequences (positions 21,565 to 24,025). Scale bar represents the number of nucleotide substitutions per site.

Using viral sequences, we classified the assigned Omicron variant into 5 subvariant groups: BA.1x (*n* = 35 participants), BA.2x (*n* = 17), BA.4/5x (*n* = 23), XBB.x (*n* = 40), and JN.x (*n* = 25) (Figure 1). Participants in the XBB.x and JN.x groups were older on average than participants in the BA.1x, BA.2x, and BA.4/5x groups (52, 54, 37, 39, and 36 years old on average, respectively, *P* = 0.043) (Table 1). Participants across the subvariant groups were similar in terms of sex, occurrence of symptoms before or during the study, and time between first positive SARS-CoV-2 test or first symptoms (whichever was earlier) and enrollment in the study. Median number of COVID-19 vaccinations increased (*P* = 0.0001) for more recent circulating subvariants, whereas days since last vaccination fluctuated over the observation period (*P* = 0.0034).

### Virologic characteristics.

**Figure 2:**
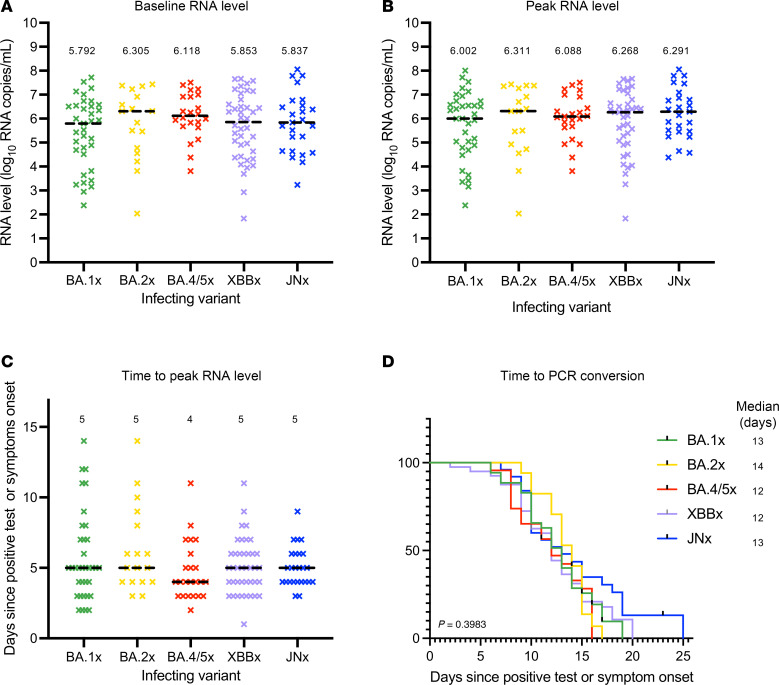
Baseline, peak, and clearance rates of SARS-CoV-2 RNA levels among Omicron subvariants. (**A**) Baseline viral RNA level (= first study nasal swab) for participants in BA.1x, BA.2x, BA.4/5x, XBB.x, and JN.x groups. *P* = 0.6371, Kruskal-Wallis test. (**B**) Peak viral RNA level for each participant in BA.1x, BA.2x, BA.4/5x, XBB.x, and JN.x groups. *P* = 0.8174, Kruskal-Wallis test. (**C**) Time to reach peak viral RNA level in days, counted from the day of first positive test or symptoms onset for each participant in BA.1x, BA.2x, BA.4/5x, XBB.x, and JN.x groups. *P* = 0.4688, Kruskal-Wallis test. (**A**–**C**) Dashed lines represent the median value for each group, which is reported above each group. (**D**) Kaplan-Meier survival curves showing time from initial positive SARS-CoV-2 test result or symptom onset until negative viral PCR for groups BA.1x, BA.2x, BA.4/5x, XBB.x, and JN.x. *P* = 0.3983, log-rank test.

**Figure 3:**
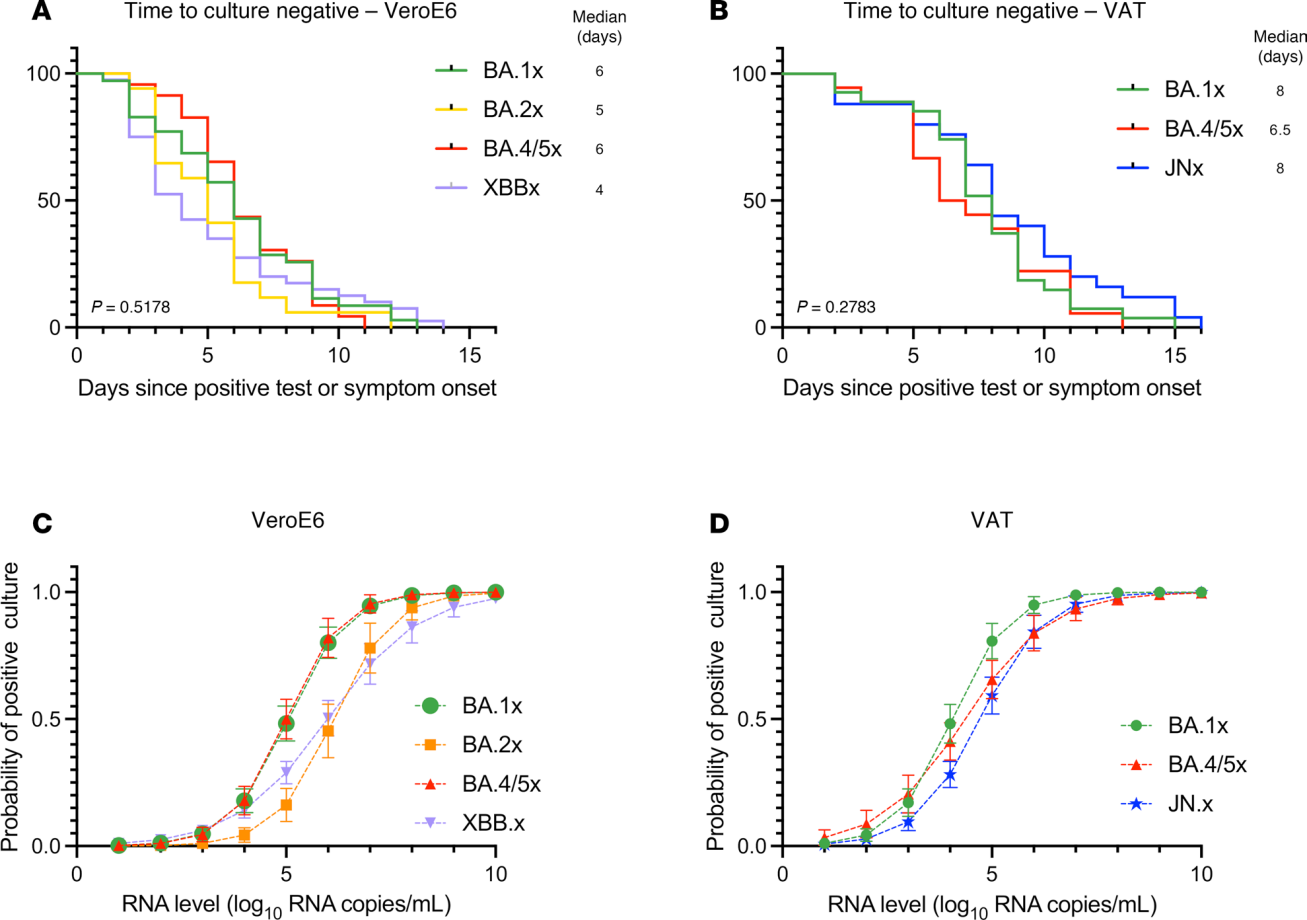
The duration of shedding live virus for Omicron subvariants. (**A**) Kaplan-Meier survival curves showing time from initial positive SARS-CoV-2 test result or symptom onset until negative viral culture on Vero-E6 cells for groups BA.1x, BA.2x, BA.4/5x, and XBB.x. *P* = 0.5178, log-rank test. (**B**) Kaplan-Meier survival curves showing time from initial positive SARS-CoV-2 test result or symptom onset until negative viral culture on VeroE6-hACE2-hTMPRSS2 (VAT) cells for groups BA.1x, BA.4/5x, and JN.x. *P* = 0.2783, Mantel-Cox log-rank test. (**C**) Logistic regression curves showing the probability (mean ± SEM) of positive viral culture on Vero-E6 cells for a range of viral RNA levels for groups BA.1x, BA.2x, BA.4/5x, and XBB.x. *P* < 0.001, Wald test; viral RNA level and variant category interaction terms from logistic regression model: BA.1x vs. BA.2x *P* = 0.004, BA.1x vs. BA.4/5x *P* = 0.871, BA.1x vs. XBB.x *P* = 0.018, BA.2x vs. BA.4/5x *P* = 0.004, XBB.x vs. BA.4/5x *P* = 0.018, and BA.2x vs. XBB.x *P* = 0.158 at 5 log_10_ copies RNA/mL. (**D**) Logistic regression curves showing the probability (mean ± SEM) of positive viral culture on VeroE6-hACE2-hTMPRSS2 cells for a range of viral RNA levels for groups BA.1x, BA.4/5x, and JN.x. *P* < 0.001, Wald test; viral RNA level and variant category interaction terms from logistic regression model: BA.1x vs. BA.4/5x *P* = 0.161, JN.x vs. BA.1x *P* = 0.051, and JN.x vs. BA.4/5x *P* = 0.545 at 5 log_10_ copies RNA/mL, JN.x vs. BA.1x *P* = 0.029 at 4 log_10_ copies RNA/mL.

We did not observe significant differences in median nasal SARS-CoV-2 RNA levels for the first sample collected (5.79–6.31 log_10_ RNA copies/mL, *P* = 0.63, Figure 2A), median peak RNA level (6.0–6.31 log_10_ RNA copies/mL, *P* = 0.82, Figure 2B), or days to reach peak RNA level (4–5 days from earliest of first positive test or symptom onset, *P* = 0.47, Figure 2C). Median days to negative PCR was similar across Omicron subvariants (12–14 days, *P* = 0.39, Figure 2D). We next assessed the median time for conversion to negative culture for all participants using a 50% tissue culture infectious dose (TCID_50_) assay. For the POSITIVES cohort, the TCID_50_ assay has previously been carried out on Vero-E6 cells (6–13). Using this established assay, median times for culture conversion were similar for BA.1x, BA.2x, BA.4/5x, and XBB.x subvariants (4–6 days, *P* = 0.52, Figure 3A). Unexpectedly, we observed a substantial discordance between nasal RNA level and viral culture for the JN.x subvariant, as JN.x samples showed no cytopathic effects on Vero-E6 cells even at high viral RNA levels. We hypothesized that JN.x may have evolved toward an alternate preferred mode of uptake into host cells. Using Vero-E6 cells overexpressing human ACE2 and TMPRSS2 (VeroE6-hACE2-hTMPRSS2, VAT), we tested the JN.x samples for cytopathic effect and were able to quantify TCID_50_ values. Using BA.1x and BA.4/5x samples, we confirmed that culture positivity on VAT cells reflected culture positivity on Vero-E6 cells at similar viral RNA levels (Supplemental Figure 2A), and that the median time for culture conversion for the BA.1x and BA.4/5x samples was not statistically different between the 2 cell lines (6–8 vs. 6–6.5 days, *P* = 0.08 and *P* = 0.23 respectively, Supplemental Figure 2B). For samples tested on VAT cells, the median times for culture conversion were 6.5–8 days for JN.x, BA.1x, and BA.4/5x (*P* = 0.28, Figure 3B). In comparing Cox proportional hazards models with and without vaccination status, we found modest increases in the hazard ratio (HR), suggesting faster time to viral culture clearance, for newer subvariants (Supplemental Table 2). This was particularly true for XBB.x, with HR increased to 1.85 (95% CI: 1.01–4.30) from 1.17 (95% CI: 0.74–1.87, *P* = 0.0045) after addition of vaccination status to the model. These results suggest that the similarity in viral clearance times seen in unadjusted models over time may partially be due to increasing immunity for more recent subvariants, which in turn may result in faster observed clearance of the virus.

We then calculated the probability of positive culture across different values of viral RNA for all studied subvariants by logistic regression. For each viral RNA level increase of 1 log_10_ unit, there was a 4-fold increase in odds ratio for a positive culture (*P* < 0.001). XBB.x and BA.2x were less likely to replicate on Vero-E6 cells at the same RNA levels (5 log_10_ RNA copies/mL) than BA.1x (*P* = 0.02 and *P* = 0.004, respectively) or BA.4/5x (*P* = 0.02 and *P* = 0.004, respectively) (Figure 3C). Similarly, JN.x was less likely than BA.1x (*P* = 0.03) to yield positive culture on VAT cells at the same RNA level (4 log_10_ RNA copies/mL) (Figure 3D). The threshold viral copy numbers for 50% probability of positive culture were lower for VAT cells than for Vero-E6 cells in both BA.1x and BA.4/5x (Supplemental Figure 2C and Supplemental Table 1).

### Symptom characteristics.

**Figure 4:**
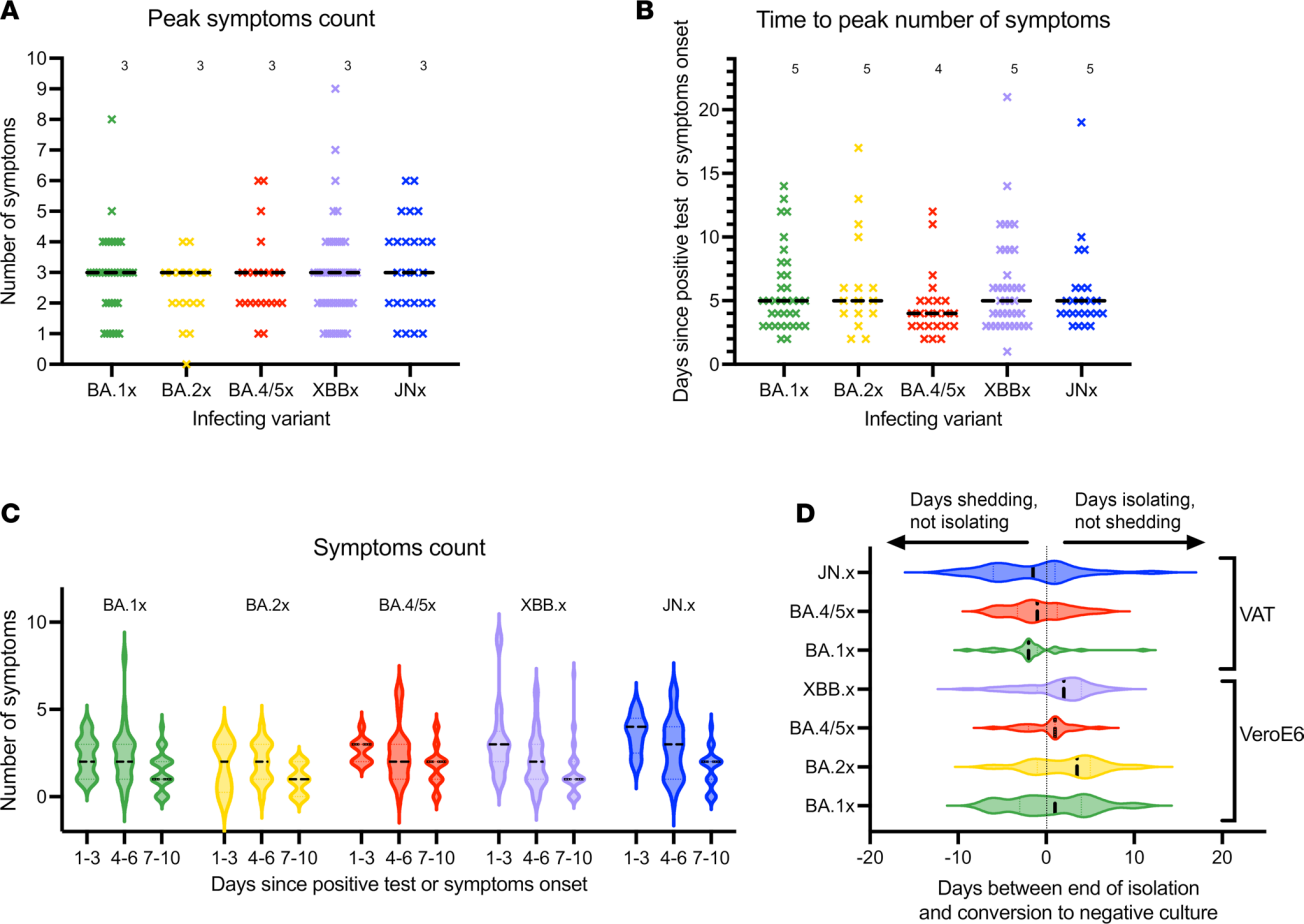
Symptom characteristics among Omicron subvariants. (**A**) Peak number of symptoms for each participant in BA.1x, BA.2x, BA.4/5x, XBB.x, and JN.x groups. *P* = 0.6454, Kruskal-Wallis test. (**B**) Time to reach peak number of symptoms in days, counted from the day of first positive test or symptoms onset for each participant in BA.1x, BA.2x, BA.4/5x, XBB.x, and JN.x groups. *P* = 0.2487, Kruskal-Wallis test. (**A** and **B**) Median values are reported above each group. (**C**) Number of symptoms at days 1–3, 4–6, and 7–10 for groups BA.1x, BA.2x, BA.4/5x, XBB.x, and JN.x. *P* = 0.2428 for days 1–3, *P* = 0.4658 for days 4–6, *P* = 0.1178 for days 7–10, Kruskal-Wallis test. (**D**) Time between end of isolation and conversion to negative viral culture for BA.1x, BA.2x, BA.4/5x, and XBB.x on Vero-E6 cells and for BA.1x, BA.4/5x, and JN.x on VAT cells. *P* = 0.2382 for Vero-E6, *P* = 0.6450 for VAT, Kruskal-Wallis test (**A**–**D**). Dashed lines represent the median value for each group.

Together with the virus and background population immunity, public health guidance has evolved over the course of the pandemic. Current guidance for respiratory viruses is based primarily on symptoms (5). We thus explored how well symptoms correlate with the virologic course of infection. All but 1 participant reported symptoms at some point during observation (Table 1). The number of peak symptoms (3 symptoms, *P* = 0.64, Figure 4A) and time to peak symptoms (4–5 days, *P* = 0.25, Figure 4B) did not differ across Omicron subvariants. When binned by days since positive test or symptom onset (1–3, 4–6 or 7–10 days), the number of symptoms also did not vary by subvariant (Figure 4C and Supplemental Figure 3). We next sought to understand what proportion of individuals are shedding replication-competent virus and thus potentially still infectious at the time they stop isolating (Figure 4D). Using current US Centers for Disease Control and Prevention (CDC) guidelines as a metric for when individuals are likely to leave isolation (5), we found that 34% (95% CI: 27.5%–42.9%) of individuals continue to shed replication-competent virus at the time that they met criteria for ending isolation for BA.1x, BA.2x, BA.4/5x, and XBB.x (based on TCID_50_ assays carried out on Vero-E6 cells, Supplemental Figure 4A). For BA.1x, BA.4/5x, and JN.x (based on TCID_50_ assays carried out on VAT cells), the proportion of participants still shedding was 64.9% (95% CI: 56.00%–77.78%). Moreover, 8.1% (95% CI: 4.35%–14.29%) of individuals continued to shed replication-competent virus at the end of the recommended masking period in the BA.1x, BA.2x, BA.4/5x, and XBB.x groups (based on Vero-E6 assays, Supplemental Figure 4B) and 19.3% (95% CI: 11.11%–32.0%) for BA.1x, BA.4/5x, and JN.x groups (based on the VAT assays). We did not find evidence that these proportions differed among subvariants. Eight-four percent of participants had 1 or more symptoms when they stopped shedding replication-competent virus, and 55% had 2 or more symptoms (Supplemental Figure 4C). Interestingly, 13 out of 140 (9.29%) participants reported no symptoms on at least one day they submitted nasal swabs containing culturable virus. Fever is a prominent feature of symptom-based guidelines (5). However, across subvariants, less than 15% of individuals reported fever at any point during the course of infection (Supplemental Figure 4D). We compared how limiting the symptoms to only cough and congestion affected the relationship between symptoms and viral shedding. Although moderately better, using only 2 symptoms did not improve the predictability of viral shedding duration based on symptoms (Supplemental Table 3).

## Discussion

In this prospective, longitudinal study of ambulatory individuals with mild to moderate COVID-19, we found that the fundamental viral dynamics of infection have not meaningfully changed across Omicron subvariants or changes in population immunity. Peak RNA, time to peak RNA, time to PCR conversion, and time to culture conversion was not different across Omicron subvariants. Notably, 28%–56% of individuals continued to shed replication-competent virus after the conclusion of the recommended isolation period.

The discordance between nasal RNA and viral culture for JN.x suggests that the JNx subvariant may have changed its preferred mode of viral uptake, as unlike all previous subvariants, we found that JN.x did not replicate in Vero-E6 cells. Comparison of BA.1x and JN.x on a Vero-E6–derived cell line overexpressing hACE2 and TMPRSS2 showed no significant difference in time to culture conversion. We hypothesize that the lower viral RNA copy number threshold for virus isolation positivity observed in VAT cells compared with Vero-E6 cells for BA.1x and BA.4/5x subvariants is, in part, due to the higher density of hACE2 receptor at the surface of VAT cells. The shift in the viral entry pathway observed for JN.x in our in vitro culture system did not translate into drastic changes in virologic characteristics in ambulatory individuals with mild infection; potential changes in tropism will need to be studied in vivo.

Similarly, our results suggest that the symptom characteristics of non-severe SARS-CoV-2 infection have not meaningfully changed across Omicron subvariants. Nonetheless, public health guidance has shifted to symptom-based isolation recommendations (5), without clear changes in available data driving those changes in recommendations. The isolation and masking guidance are partially based on fever. Less than 20% of participants in this study experienced fever at any point, raising the question of whether fever offers a useful criterion for making isolation decisions.

Shedding replication-competent virus from the nasopharynx is a reasonable proxy for infectiousness (15). Our data on duration of shedding of viable virus suggest that current symptom-based criteria are not a good indicator of when individuals are no longer potentially infectious. For this cohort, isolation guidance is poorly correlated with infectiousness in both directions; some individuals would be isolating longer than they are likely to be infectious, while others would leave isolation while still robustly shedding replication-competent virus and thus likely infectious. For example, over 50% of individuals infected with the most recent subvariants are still shedding replication-competent virus at the time they meet criteria to end isolation. Conversely, 84% of people with symptoms cease to shed virus before their symptoms resolve. These results are consistent with our previous study of individuals with and without nirmatrelvir-ritonavir–associated rebound, in which we found that symptoms were not consistently correlated with shedding replication-competent virus (8) and consistent with studies by others demonstrating discordance between symptoms and shedding replication-competent virus (16). These findings raise important questions about whether alternate criteria for ending isolation, such as reversion of antigen testing, could more effectively prevent forward viral transmission.

There are some limitations to our study. The study population included ambulatory individuals with mild infection in the greater Boston area, so findings may not be generalizable to all populations. We excluded individuals who were immunosuppressed, were hospitalized with SARS-CoV-2 infection, or taking antivirals. A small number of participants (*n* = 11) were excluded due to missing swab or other data. With the changes in testing requirements over time, we did not capture as many asymptomatic infections as in the early days of this study, leading to an underestimation of mild cases. Additionally, the effect of background immunity due to increasing accumulation of prior infections could not be evaluated. In this study, prior infections were self-reported and not all participants consented to blood collection, preventing the use of anti–SARS-CoV-2 Nucleocapsid titers as a proxy for prior infection. Nonetheless, if increasing background immunity is partially responsible for reduced shedding in later variants, the observed effect remains that virologic shedding by and large has remained similar, which is of public health importance.

Because individuals with severe disease and/or immunosuppression tend to shed virus for longer periods (9, 17, 18), our results may underestimate viral shedding duration in these other populations. Sample sizes were in each subvariant group were relatively small (*n* ranging from 17–40). Finally, while the capacity to culture live virus from nasal samples is the best available lab-based proxy for infectiousness, to date there are few data to definitively link any particular RNA level or viral culture quantification with transmission (15).

In summary, we demonstrate that Omicron subvariants have had a limited impact on viral dynamics and symptoms in a population cohort of SARS-CoV-2 infection.

## Methods

### Sex as a biological variable.

POSITIVES enrolls both male and female participants, and findings from this analysis were similar for both sexes.

### Study design.

POSITIVES is an ongoing prospective cohort study of individuals with acute COVID-19 within the Mass General Brigham health care system. Adult outpatients diagnosed with COVID-19 were identified through automated medical record review or referrals and recruited as soon as possible after a positive SARS-CoV-2 test.

### Cohort and sample collection.

Out of 397 participants enrolled between December 2021 and September 2024 in the cohort, 300 were eligible for this analysis (97 participants excluded for inpatient status or immunosuppression treatment). A further 160 participants were excluded because they did not provide enough samples (3 or less), they received antiviral treatment, or the infecting variant could not be confirmed as Omicron, leading to an analytic cohort of 140 participants.

Anterior nasal (AN) swab samples were self-collected and stored in viral transport medium 3 times a week for the first 2 weeks following study enrollment and then weekly thereafter until participants had persistently undetectable viral RNA levels. Samples were then transported to the processing laboratory where viral transport medium containing anterior nasal swabs were aliquoted and stored at –80°C until testing. At enrollment and on each date of swab collection, participants completed a survey on acute COVID-19 symptoms, including presence (yes or no) and severity (mild, moderate, or severe) of cough, fever, muscle aches, fatigue, sore throat, shortness of breath, loss of taste, loss of smell, congestion, and headache.

### Cell lines and reagents.

Vero-E6 (ATCC) and VeroE6-hACE2-hTMPRSS2 (BEI Resources) cell lines were maintained in DMEM (Corning) supplemented with HEPES (Corning), 1× penicillin (100 IU/mL)/streptomycin (100 μg/mL) (Corning), 1× glutamine (GlutaMax, Thermo Fisher Scientific), and 10% FBS (MilliporeSigma).

### Whole-genome sequencing.

We performed whole-genome sequencing, as previously reported (8, 13, 19), using the Illumina COVIDSeq Test protocol. Libraries were constructed using the Illumina Nextera XT Library Prep Kit, then pooled and quantified using a Qubit High Sensitivity dsDNA kit. The size and concentrations of the DNA libraries were determined on an Agilent TapeStation 4150 using the High Sensitivity D1000 ScreenTape assay. Genomic sequencing was then performed on an Illumina NextSeq 2000, Illumina NextSeq 550, or Illumina NovaSeq SP instrument. Sequences with an assembly length greater than 24,000 base pairs were considered complete genomes, and we assigned those sequences a Pango lineage. All sequences were deposited in GenBank and GISAID. The samples were submitted to NCBI with BioProject accession numbers PRJNA759255 and PRJNA1261936.

### Spike-specific gene next-generation sequencing.

The target gene next-generation sequencing (NGS) was carried out as previously reported (14). AN swab samples were used for RNA extraction, and cDNA was synthesized using Superscript IV reverse transcriptase (Invitrogen) following the manufacturer’s instructions. Spike genes were amplified with a nested PCR approach, using in-house–designed primers. NGS was performed using the Illumina MiSeq platform.

### Maximum likelihood phylogenetic tree.

SARS-CoV-2 spike sequences (positions 21,565 to 24,025) were compiled in MEGA 11 (20) and aligned relative to the Wuhan-Hu-1 reference sequence (NCBI Reference Sequence: NC_045512.2) using MAFFT (21). The best-fit maximum likelihood model was evaluated using IQTree’s ModelFinder tool (22). The model with the lowest Bayesian information criterion was TPM3u + F + I (23), which was run using IQTree’s Tree Inference tool (24, 25). The consensus tree generated from 1000 bootstraps was visualized using FigTree v1.4.4 (26). The phylogenetic tree generally includes the earliest SARS-CoV-2 spike sequence available per participant.

### Viral RNA level quantification.

Viral RNA level quantification was conducted as previously reported (7, 8, 13, 19). SARS-CoV-2 viral RNA was isolated from the aliquoted viral transport media and tested with a qPCR assay using the US CDC 2019 nCoV_N1 primer and probe set and quantified using a standard curve.

### Viral culture.

We performed viral culture in the BSL3 laboratory of the Ragon Institute of Mass General Brigham, MIT, and Harvard as previously reported (6–9, 12–14, 19, 27). Briefly, AN samples were filtered through 0.45-μm filters and used to inoculate cells seeded in 96-well plates in media supplemented with polybrene (Santa Cruz Biotechnology). After a 1-hour spin at 2,000*g*, the plates were incubated at 37°C and 5% CO_2_. The plates were evaluated for cytopathic effect 7 days after infection on a bright-field microscope and TCID_50_/mL titers were calculated using the Reed-Muench method.

### Statistics.

Prism version 10.2.0 (GraphPad) was used to generate all graphs, and statistical analysis was carried out using Prism or Stata/SE 17.0 (Statacorp). A *P* value of less than 0.05 was considered significant. All collected demographics and experimental data from POSITIVES were managed using REDCap (Research Electronic Data Capture) electronic data capture tools hosted at https://redcap.partners.org/redcap/ (28, 29). Subgroup analytic datasets were then generated for further analysis. Categorical cohort characteristics parameters were assessed using Pearson’s χ^2^ tests, continuous parameters were assessed using Kruskal-Wallis tests. For virologic and symptoms characteristics analysis, Kruskal-Wallis tests were used to compare the medians of multiple groups. For survival analysis, we used the Kaplan-Meier method to estimate the survivor function for time to negative viral PCR and time to negative viral culture and applied log-rank (Mantel-Cox) test. For these analyses, we defined the date of conversion to negative as the mean between the first negative value and the immediate prior positive value. For participants with no positive culture results during observation, we defined the culture conversion date as the mean between the date of diagnosis or first symptoms and the date of the first study sample collection. All time-related outcomes are reported as “days since positive test or symptom onset”; we took the time of symptom onset or time of first positive SARS-CoV-2 test (whichever was earliest) as the origin of the timescale and designated as “day 0.” For the samples that were tested on 2 cell lines in the TCID_50_ assay, we used simple linear regression to assess whether the cell line used changed the outcome of the viral culture assay. To assess the relationship between viral RNA level and presence of replication-competent virus, we applied logistic regression with the robust or sandwich estimator of variance to calculate a probability of positive culture across different values of viral RNA level for all studied subvariants, including the full dataset. We used the logistic regression model to compare the likelihood of a positive culture at a viral RNA level of 5 log_10_ copies RNA/mL and then we compared the relationship between viral RNA level and the subvariant categories using interactions terms. We also calculated the viral RNA copy number for a 50% probability of virus isolation by sigmoidal 4PL interpolation of the logistic regression. Because of temporal differences in the emergence of individual subvariants, we considered whether the numbers of prior vaccinations received might confound the relationship between subvariants and viral clearance. To address this, we fit Cox proportional hazards models with time to reach culture conversion as the outcome, subvariant as primary exposure and with and without prior vaccination status. Vaccination status was categorized into similarly sized groups of 2 or fewer, 3, and 4 or more vaccinations. We calculated an “end of isolation” and “end of masking” date for each participant as per CDC guidelines (5), taking into account symptoms improvement and no fever for 24 hours without any antipyretic intervention for isolation and adding 5 days for masking. We then compared the end of isolation date with the date of conversion to negative culture and categorized participants as shedding virus at end of isolation or isolating while not shedding. Since fever is a distinct symptom criterion in the CDC guidelines, we also categorized participants into 2 groups: experienced fever or did not experienced fever during the study period. χ^2^ tests of proportions were used to compare proportions between the groups. Finally, we categorized the participants into 7 groups according to how many symptoms they experienced on the day they converted to a negative viral culture (0–6 symptoms) and calculated the cumulative proportion of participants experiencing symptoms on the day of culture conversion (i.e., day they stopped shedding virus). All subvariant groups were combined for this analysis.

### Study approval.

Study procedures were reviewed and approved by the Human Subjects IRB at Mass General Brigham under protocol no. 2021P000812 and BSL3 laboratory work was approved by the Institutional Biosafety Committee. All participants gave verbal informed consent, as written consent was waived by the review committee based on the risk/benefit ratio of requiring in-person interactions for an observational study of COVID-19.

### Data availability.

All supporting de-identified data are included in the Supporting Data Values file. STATA analytic code will be made available upon request.

## Author contributions

JB conducted experiments, participated in the data analysis, and drafted the initial manuscript. OTG, CM, GEE, MCC, YL, and BML conducted experiments and participated in the data analysis. ZR participated in data collection and managed data collection. KS, DT, and CBM participated in data collection. EP managed data collection and participated in the data analysis. AA, EA, MB, and DG conducted experiments. TDV participated in data collection. JMV conceived the study design and provided reagents. JEL and JZL conceived the study design, provided reagents, and participated in the data analysis. MJS conceived the study design and participated in the data analysis. AKB conceived the study design, provided reagents, participated in the data analysis, and drafted the initial manuscript. All authors contributed editorial input and approved the final version of the manuscript.

## Funding support

This work is the result of NIH funding, in whole or in part, and is subject to the NIH Public Access Policy. Through acceptance of this federal funding, the NIH has been given a right to make the work publicly available in PubMed Central.

- NIH, grants U19 AI110818 and R01 AI176287.
- NIH, grant K24 HL166024, to MJS.
- Massachusetts Consortium on Pathogen Readiness (MassCPR) SARS-CoV-2 Variants Program.
- Massachusetts General Hospital Department of Medicine.

## Supplementary Material

Supplemental data

ICMJE disclosure forms

Supporting data values

## Figures and Tables

**Table 1:**
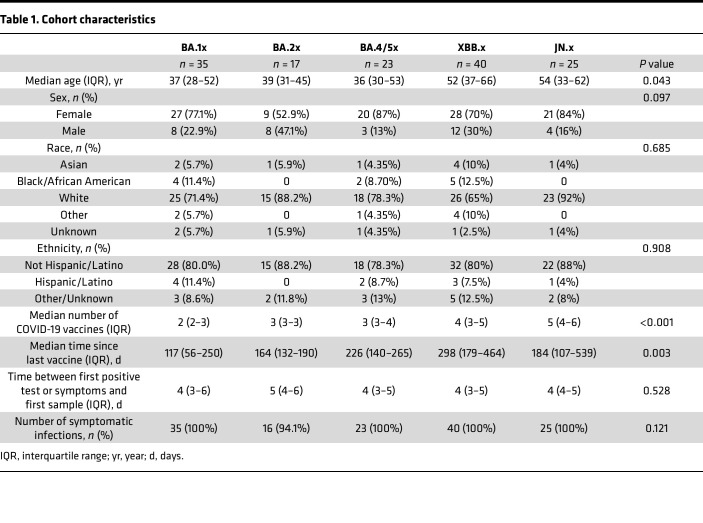
Cohort characteristics

## Version 1 09/09/2025

In-Press Preview

## Version 2 10/22/2025

Electronic publication
