# Supplementary material for: Virologic characteristics of SARS-CoV-2 infection across evolving Omicron subvariants
Source: JCI Insight. 2025 Sep 9;10(20):e192228. doi: 10.1172/jci.insight.192228 (PMC12581670; doi:10.1172/jci.insight.192228)
Supplement: Supplemental data [file jciinsight-10-192228-s268.pdf]

Supplemental Material

Virologic characteristics of SARS-CoV-2 infection across evolving Omicron subvariants.

Authors:

Julie Boucau, Owen T. Glover, Caitlin Marino, Gregory E. Edelstein, Manish C. Choudhary, Yijia Li, Brooke M. Leeman, Zahra Reynolds, Karry Su, Dessie Tien, Chase B. Mandell, Eliza Passell, Andrew Alexandrescu, Emory Abar, Mamadou Barry, Dibya Ghimire, Tammy D. Vyas, Jatin M. Vyas, Jacob E. Lemieux, Jonathan Z. Li, Mark J. Siedner, Amy K. Barczak

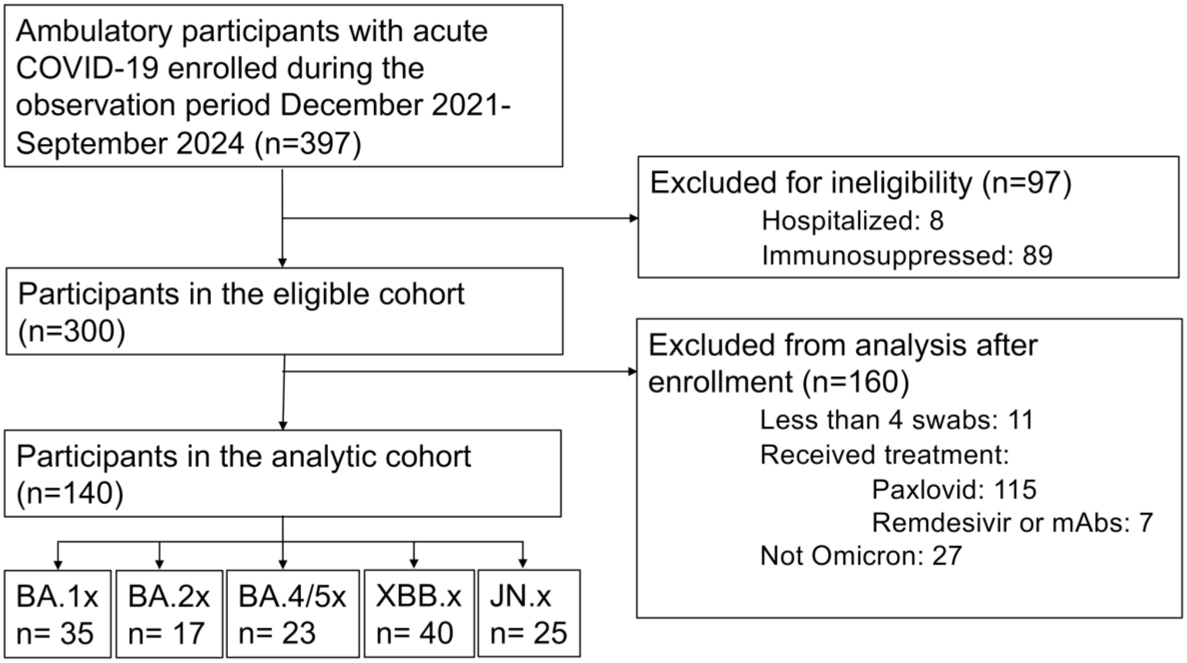

Supplemental Figure 1. Study flow diagram

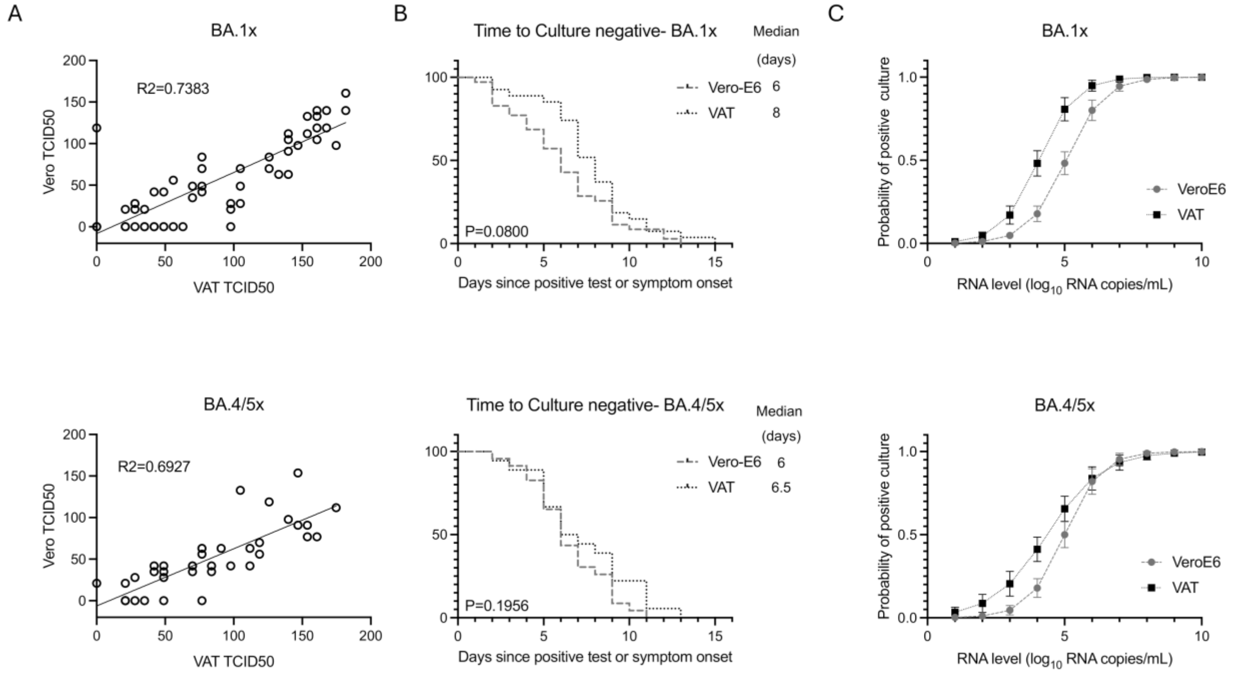

Supplemental Figure 2. Comparison of culture-based virological characteristics for assays carried out on Vero-E6 and VeroE6-hACE2-hTMPRSS2 (VAT) cells. **A.** Correlation of 50% tissue culture infectious dose (TCID50) values obtained from viral culture on Vero-E6 cells (y-axis) and VAT (x-axis) cells from nasal swabs from BA.1x (top) and BA.4/5x (bottom) infected participants. For BA.1x slope = 0.7352, R^2=0.7383 and for BA.4/5x slope = 0.6882, R^2=0.6927, simple linear regression. **B.** Kaplan–Meier survival curves showing time from initial positive SARS-CoV-2 test result or symptom onset until negative viral culture on Vero-E6 and VAT cells for groups BA.1x (top) and BA.4/5x (bottom). P=0.0800 for BA.1x and P=0.1956 for BA.4/5x, log-rank test. **C.** Logistic regression curves showing the probability (mean and SEM) of positive viral culture on Vero-E6 and VAT cells for a range of viral RNA levels for groups BA.1x (top), and BA.4/5x, (bottom). For BA.1x $P<0.001$, Wald test; viral RNA level and cell line interaction terms from logistic regression model for BA.1x VeroE6 vs. VAT P=0.017 at 5 $\log_{10}$ copies RNA/mL and for BA.4/5x VeroE6 vs. VAT P=0.721 at 4 $\log_{10}$ copies RNA/mL.

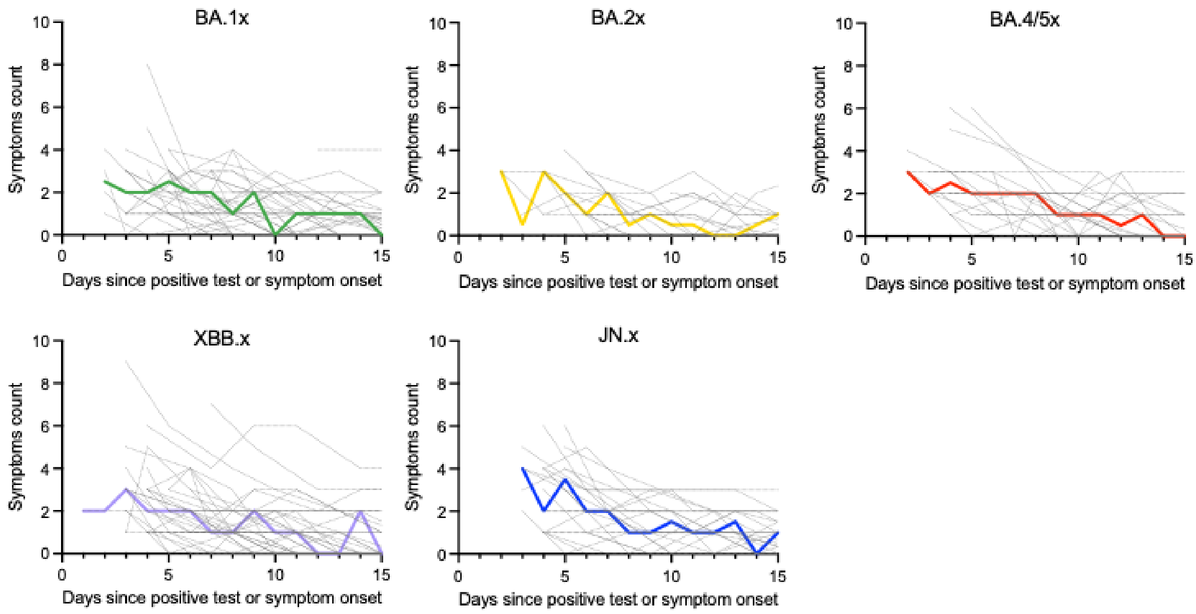

Supplemental Figure 3. Symptom count over time for each participant in the Omicron subvariant groups BA.1x, BA.2x, BA.4/5x, XBB.x and JN.x. The daily median count of symptoms for each subvariant is shown in bolded color.

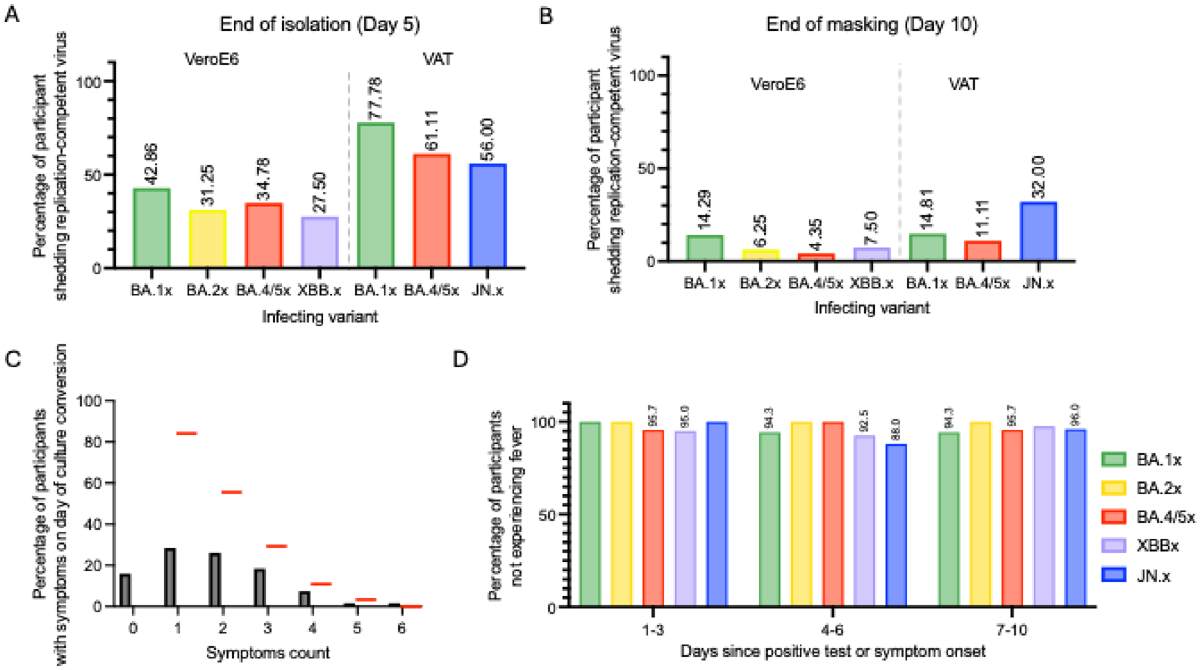

Supplemental Figure 4. Relationship between symptoms and virus shedding. **A.** Percentage of participants in BA.1x, BA.2x, BA.4/5x, XBB.x and JN.x groups who are shedding replication-competent virus at the end of the recommended isolation (Day 5) per CDC guidelines based on TCID50 assay carried out on Vero-E6 (left) or VAT (right). P=0.5665 for Vero-E6, P=0.2276 for VAT, Chi-square proportion test. **B.** Proportion of participants in BA.1x, BA.2x, BA.4/5x, XBB.x and JN.x groups who are shedding replication-competent virus at the end of the recommended masking period (Day 10) per CDC guidelines based on TCID50 assay carried out on Vero-E6 (left) or VAT (right). P=0.5519 for VeroE6, P=0.1660 for VAT, Chi-square proportion test. **C.** Percentage of participants experiencing symptoms on the day they stopped shedding replication-competent virus. Grey bars represent the percentage of participants experiencing 0, 1, 2, 3, 4, 5, 6 symptoms while red lines represent the cumulative percentage of participants experiencing 1 or more, 2 or more, 3 or more symptoms at the end of isolation. **D.** Percentage of participants who did not experience fever at days 1-3, 4-6 and 7-10 in BA.1x, BA.2x, BA.4/5x, XBB.x and JN.x groups. P=0.5426 for Days 1-3, P=0.4046 for Days 4-6, P=0.9624 for Days 7-10, Chi-square proportion test.

Supplemental Table 1: Viral RNA copy number for 50% probability of positive viral culture for each Omicron subvariant.

	**BA.1x**	**BA.2x**	**BA.4/5x**	**XBB.x**	**JN.x**
Vero-E6 cell line	5.047	6.130	5.001	5.983	N.A.
VAT cell line	4.049	N.A.	4.356	N.A.	4.714

VAT: Vero-hACE2-hTMPRSS2, N.A.: not applicable.
Viral RNA copy number reported as $\log_{10}$ RNA copy number per mL. The values were obtained by logistic regression (sigmoidal, 4PL interpolation).

Supplemental Table 2: Relationship of time to viral clearance, infecting Omicron subvariants, and vaccination status as shown by Hazard Ratios and 95% Confidence Intervals.

		Model without Vaccination Status			Model with Vaccination Status		
	Subvariant	Hazard Ratio	95% CI	P-value	Hazard Ratio	95% CI	P-value
Vero-E6	BA.1x	REF	--		REF	--	
	BA.2x	1.38	0.77-2.49	0.27	1.37	0.75-2.51	0.30
	BA.4/5x	0.98	0.58-1.66	0.93	1.18	0.66-2.12	0.58
	XBB.x	1.17	0.74-1.87	0.50	1.85	1.01-3.40	0.045
VAT	BA.1x	REF	--		REF	--	
	BA.4/5x	1.10	0.61-2.01	0.75	1.36	0.64-2.93	0.43
	JN.x	0.73	0.42-1.28	0.27	1.02	0.47-2.22	0.96

CI: Confidence Interval.

Cox proportional hazards models were used to calculate Hazard Ratios and CIs.

Supplemental Table 3. Comparison of symptoms and viral shedding duration using the full list of symptoms or limiting the analysis to cough and congestion only.

	**Shedding stops first**	**Symptoms stop first**	**Same day**
10 symptoms	93 (80.57%)	9 (8.11%)	9 (8.11%)
Cough and congestion	90 (75.63%)	22 (18.48%)	7 (5.88%)

The number of participants in each category and percentages are reported.
